# MWASTools: an R/bioconductor package for metabolome-wide association studies

**DOI:** 10.1093/bioinformatics/btx477

**Published:** 2017-07-26

**Authors:** Andrea Rodriguez-Martinez, Joram M Posma, Rafael Ayala, Ana L Neves, Maryam Anwar, Enrico Petretto, Costanza Emanueli, Dominique Gauguier, Jeremy K Nicholson, Marc-Emmanuel Dumas

**Affiliations:** 1 Department of Surgery and Cancer, Computational and Systems Medicine, Imperial College London, UK; 2 Division of Myocardial Function, National Heart and Lung Institute, Imperial College London, UK; 3 Duke-NUS Medical School, Singapore; 4 Bristol Heart Institute, University of Bristol, UK

## Abstract

**Summary:**

MWASTools is an R package designed to provide an integrated pipeline to analyse metabonomic data in large-scale epidemiological studies. Key functionalities of our package include: quality control analysis; metabolome-wide association analysis using various models (partial correlations, generalized linear models); visualization of statistical outcomes; metabolite assignment using statistical total correlation spectroscopy (STOCSY); and biological interpretation of metabolome-wide association studies results.

**Availability and implementation:**

The MWASTools R package is implemented in R (version  > =3.4) and is available from Bioconductor: https://bioconductor.org/packages/MWASTools/.

**Supplementary information:**

Supplementary data are available at *Bioinformatics* online.

## 1 Introduction

Owing to sustained developments in high-throughput platforms [i.e. nuclear magnetic resonance (NMR) spectroscopy and mass spectrometry (MS)], metabolic phenotyping (metabotyping) is now used for large-scale epidemiological applications such as metabolome-wide association studies (MWAS) (Elliott *et al.*, 2015; Holmes *et al.*, 2008; Nicholson *et al.*, 2002). Customized statistical modeling approaches and data visualization tools are essential for biomarker discovery in large-scale metabotyping studies. Several software packages were developed to detect and visualize metabolic changes between conditions of interest using multivariate statistical methods (Gaude *et al.*, 2013; Thevenot *et al.*, 2015). However, a major limitation of these multivariate models from an epidemiological perspective is that these do not properly account for confounding factors, which might distort the observed associations between the metabolites and the condition under study (Elliott *et al.*, 2015).

Here, we present an R package to perform MWAS using univariate hypothesis testing with efficient handling of epidemiological confounders (Elliott *et al.*, 2015). Our package provides a versatile and user-friendly MWAS pipeline with a number of functionalities, ranging from quality control (QC) analysis of metabonomic data to visualization and biological interpretation of MWAS analysis results.

## 2 Methods and features

The MWASTools package is organized in four functional modules: (i) QC analysis; (ii) MWAS analysis; (iii) visualization of MWAS results; (iv) metabolite assignment using correlation analysis. For demonstration purposes, the MWASTools package was used to analyse plasma ^1^H NMR metabolic profiles of 506 patients from the FGENTCARD cohort (Rodriguez-Martinez *et al.*, 2017b).

### 2.1 QC analysis

MWASTools performs essential QC analyses via Principal Component Analysis (PCA) and by computing the coefficients of variation (CV) (ratio of standard deviation to the mean) of individual metabolic features across the QC samples (Dumas *et al.*, 2006). The results from PCA are visualized using score plots, where tight clustering of the QC samples indicates good overall reproducibility (Supplementary Fig. S1A). The results from CV analysis can be visualized with different plots: (i) a histogram showing the distribution of CVs across the metabolic features; (ii) an NMR spectrum colored based on the CV of each spectral signal (Supplementary Fig. S1B); or a MS-based scatter plot colored based on the CV of each MS feature (Supplementary Fig. S2). MWASTools also allows filtering the metabolic variables based on a given CV threshold.

### 2.2 MWAS analysis

Following QC analysis, MWASTools tests for association between the phenotype under investigation [e.g. type II diabetes (T2D)] and each metabolic feature (or metabolite). Depending on the nature of data to be modeled, the user can choose among the following association methods: linear/logistic regression or Pearson/Spearman/Kendall correlation. The models can be adjusted for confounder factors, including age, gender or other clinical covariates (e.g. medication). The *P-*values are corrected for multiple-testing with several possible methods, such as Benjamini–Hochberg (BH) procedure (Benjamini and Hochberg, 1995). MWASTools allows performing model validation through non-parametric bootstrapping. Finally, MWAS analysis results can be filtered according to a given significance threshold.

### 2.3 Visualization of MWAS results

**Fig. 1:**
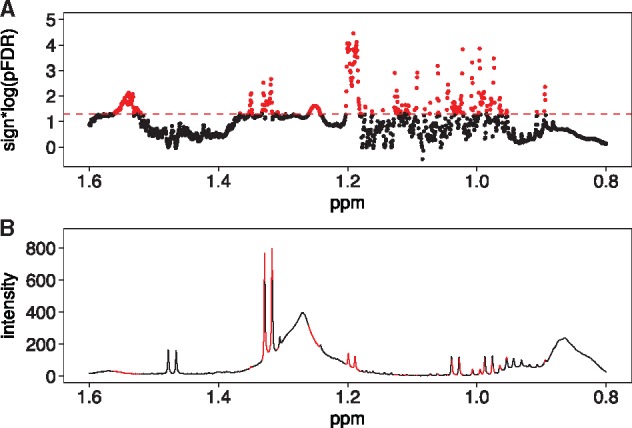
Visualization of the associations of T2D with plasma ^1 ^H NMR metabolites in the FGENTCARD cohort (*n* = 506). The associations were computed using logistic regression adjusted for age, gender and body mass index. (**A**) Partial skyline plot (δ 0.80–1.60) showing the −log_10_ (pFDR) × sign of beta coefficient of each NMR signal. Statistically significant signals positively associated with T2D were colored in red. (**B**) NMR spectrum (δ 0.80–1.60) of a QC sample colored based on association results (Color version of this figure is available at *Bioinformatics* online.)

MWASTools provides a series of customizable tools to visualize the results from MWAS analysis. For NMR data, MWASTools generates a skyline plot, where the chemical shifts are displayed along the x-axis and the log10 of the *P*-values (sign adjusted for the direction of the association) are displayed on the y-axis (Fig. 1). For other types of metabonomic data, the results are represented using: an analogous bar plot (Supplementary Fig. S4A); a MS-based scatter plot **(**Supplementary Fig. S3); a correlation-based metabolic network (Supplementary Fig. S4B); or a heatmap (Supplementary Fig. S5). Finally, the metabolites identified by MWAS can be mapped onto biological pathways (Kanehisa and Goto, 2000), and visualized using pathway-based or shortest path-based networks (Posma *et al.*, 2014; Rodriguez-Martinez *et al.*, 2017a; Shannon *et al.*, 2003) (Supplementary Figs S6–S7).

### 2.4 Structural assignment of NMR features

MWASTools performs statistical total correlation spectroscopy (STOCSY) analysis (Cloarec *et al.*, 2005) to facilitate the assignment of NMR variables significantly associated with the phenotype under study. The results are represented in a pseudo-NMR spectrum displaying the covariance (height) and Pearson/Spearman correlation coefficient (color) of all NMR variables with the variable of interest (driver) (Supplementary Figure S8). In order to highlight intramolecular correlation patterns, only NMR variables significantly correlated with the driver signal are shown.

## 3 Discussion

Altogether, the MWASTools R package provides an integrated pipeline with efficient analysis and visualization tools for: (i) performing QC analysis; (ii) conducting robust MWAS analysis with efficient handling of epidemiological confounders; (iii) structural assignment of metabolic features of interest; (iv) biological interpretation of MWAS results. The MWASTools package can be applied to both targeted and untargeted metabonomic datasets, acquired with different analytical platforms. The open nature of R allows for integration of MWASTools with other packages for the analysis of metabonomic data.

## Funding

This work was supported by Medical Research Council Doctoral Training Centre scholarship (MR/K501281/1), Imperial College scholarship (EP/M506345/1), La Caixa studentship to A.R.M.; FCT(BD/52036/2012 to A.L.N.); British Heart Foundation program grant (RG/15/5/31446) to C.E. and E.P.; BHF Chair to CE (CH/15/31199); European Commission (FGENTCARD, LSHG-CT-2006-037683 to D.G., J.K.N. and M.E.D.

*Conflict of Interest*: none declared.

## Supplementary Material

Supplementary Data
