# Supplementary material for: MWASTools: an R/bioconductor package for metabolome-wide association studies
Source: Bioinformatics. 2017 Jul 26;34(5):890–2. doi: 10.1093/bioinformatics/btx477 (PMC6049002; doi:10.1093/bioinformatics/btx477)
Supplement: Supplementary Data [file btx477_arm_si_bioinf20170309r1_180617.pdf]

MWASTools: an R/Bioconductor package for metabolome-wide association studies

Andrea Rodriguez-Martinez[1], Joram M. Posma[1], Rafael Ayala[1], Ana L. Neves[1], Maryam Anwar[2], Enrico Petretto[3], Constanza Emanueli[2,4], Dominique Gauguier[1,5], Jeremy K. Nicholson[1] and Marc-Emmanuel Dumas[1*]

[1]Computational and Systems Medicine, Department of Surgery and Cancer, Faculty of Medicine, Imperial College London, London UK.

[2]Division of Myocardial Function, National Heart and Lung Institute, Imperial College London, U.K.

[3]Duke-NUS Medical School, Republic of Singapore.

[4]Bristol Heart Institute, University of Bristol, UK.

[5]Sorbonne Universities, University Pierre & Marie Curie, University Paris Descartes, Sorbonne Paris Cité, INSERM UMR_S 1138, Cordeliers Research Centre, Paris, France.

Supplementary material

In this document, we provide the following information:

- Instructions for how to install and get started with MWASTools.
- Several figures illustrating some of the main functionalities of MWASTools. All figures were generated using plasma ^{1}H NMR metabolic profiles from the FGENTCARD cohort (n = 506) and simulated LC-MS data.

A reference manual describing all the functionalities of MWASTools, and a vignette containing a common workflow for how the package can be used, can be found at: https://bioconductor.org/packages/MWASTools/

Installation instructions

MWASTools is currently available from Bioconductor (>= 3.5). The devel version of the package contains the latest updates, while the release version is a more stable version. For details, check: https://bioconductor.org/packages/MWASTools/

To install MWASTools, start R (or RStudio) and follow the instructions shown below:

```
# 1) Install BiocInstaller and BiocGenerics
source("https://bioconductor.org/biocLite.R")
biocLite(c("BiocInstaller", "BiocGenerics"))

# 2.A) Install the release version of MWASTools
source("https://bioconductor.org/biocLite.R")
biocLite("MWASTools")

# 2.B) Install the devel version of MWASTools
source("https://bioconductor.org/biocLite.R")
BiocInstaller::useDevel()
biocLite("MWASTools")

# 3) Remove package
```

To remove the package use: remove.packages("MWASTools") and restart R.

Get started

```
# Under R, and after having installed MWASTools, load the package using:
library(MWASTools)

# To see the vignette of the package use:
browseVignettes("MWASTools")

# To get help with any function (e.g. MWAS_stats) use:
help("MWAS_stats")
```

Data format

MWASTools is integrated with the SummarizedExperiment package, which allows a more efficient handling of metabolic and clinical datasets and avoids sample mismatching. Thus, most MWASTools functions require as input a SummarizedExperiment object, which can be generated using the “MWAS_SummarizedExperiment” function. This function requires three input arguments:

- **metabo_matrix:** numeric matrix containing the metabolic data (*e.g.* NMR peak intensities or metabolite concentrations). The columns of this matrix must correspond to the metabolic variables and the rows to the samples. Column and row names must contain the metabolite identifiers (ids) (*e.g.* chemical shifts for NMR data) and the sample ids, respectively. An example is shown below:

	0.401	0.402	...	9.998	9.999
P1	0.37	0.39	...	0.38	0.42
P2	0.44	0.41	...	0.37	0.43
:	:	:	...	:	:
QC9	0.43	0.41	...	0.36	0.44
QC10	0.45	0.42	...	0.39	0.41

- **clinical_matrix**: numeric matrix containing the clinical data. The columns of the matrix must correspond to the phenotypic variables and the rows to the samples. Column and row names must contain the phenotype ids and the sample ids, respectively. For samples without clinical data (*e.g.* quality control (QC) samples), NA values must be used. An example is shown below:

	Age	Gender	...	T2D	BMI
P1	50	0	...	1	24
P2	44	1	...	0	33
:	:	:	...	:	:
QC9	NA	NA	...	NA	NA
QC10	NA	NA	...	NA	NA

- **sample_type:** numeric vector indicating sample type (*i.e.* experimental sample or QC sample). The vector must be coded as follows: experimental sample = 0, QC sample = 1. If QC samples are not available, all the elements of this vector must be 0.

A

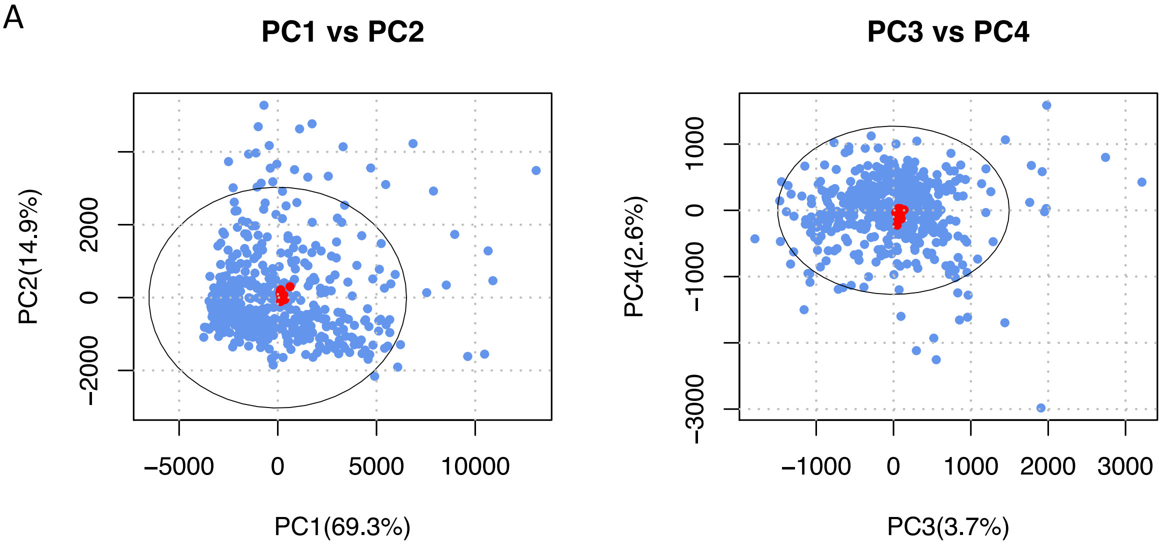

B

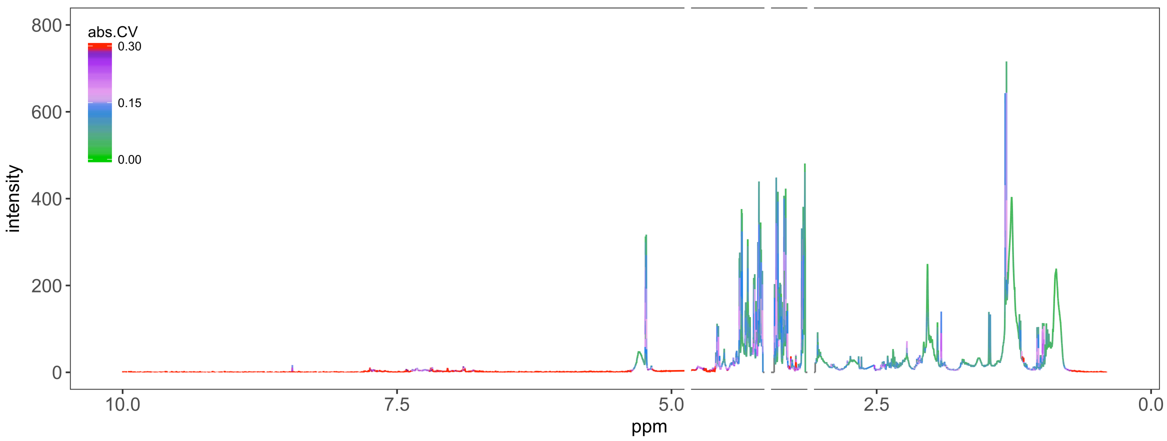

Figure S1. Visualization of quality control (QC) analysis using 506 plasma ^{1}H NMR metabolic profiles from the FGENTCARD cohort. (A) Score plots resulting from PCA analysis on mean centered data. The QC samples (n = 10), colored in red, appeared tightly clustered in the center of the Hotelling's ellipse, indicating good overall analytical reproducibility. (B) NMR spectrum of a QC sample colored according to the coefficient of variation of each NMR signal, using a CV threshold of 0.30. Most of the NMR signals corresponding to actual metabolites exhibited CV < 0.30, further confirming the reproducibility and stability of the analytical run.

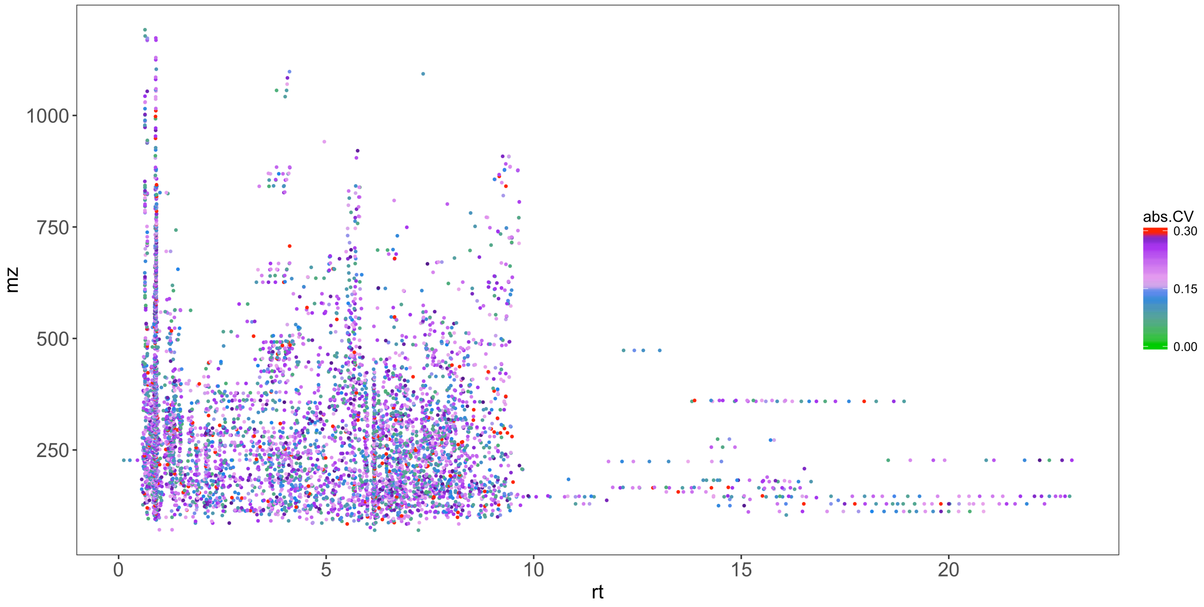

Figure S2. Visualization of quality control (QC) analysis results using simulated LC-MS data. The metabolic features are represented in dots colored according to their CV values. Metabolic features with CV equal or above 0.30 were colored in red.

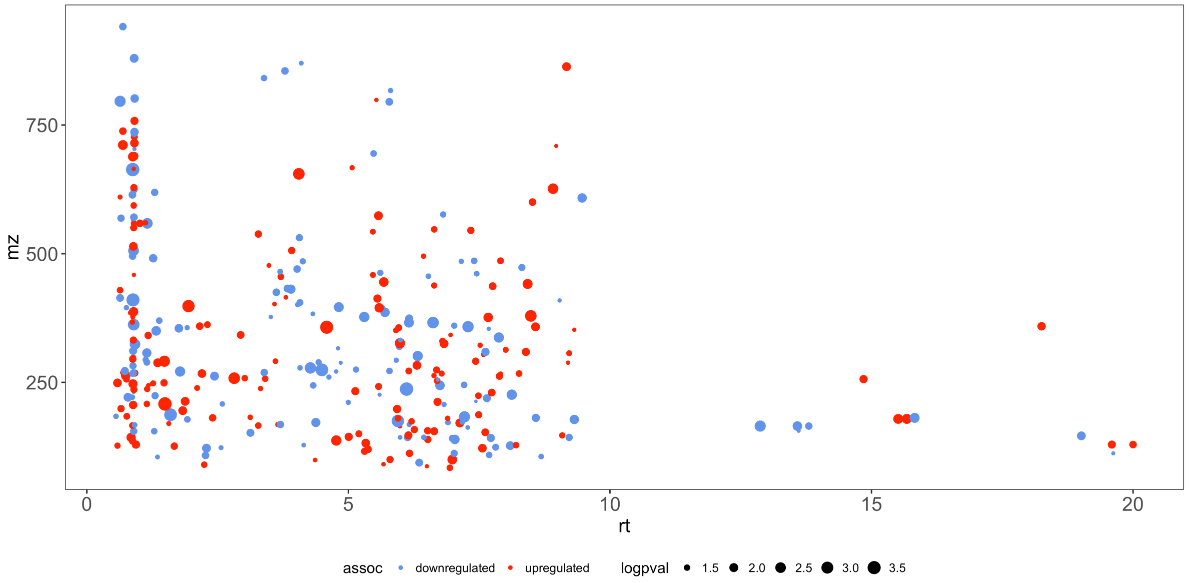

Figure S3. Visualization of MS-based MWAS results using simulated LC-MS data. The dots of the scatter plot represent significant metabolic-features, based on a significance threshold of 0.05 after adjusting for multiple-testing. The color of the dots represents the direction of the association (blue: downregulation and red: upregulation) and the size of the dots indicates the strength of the association (*i.e.* - log10(*p*-value)).

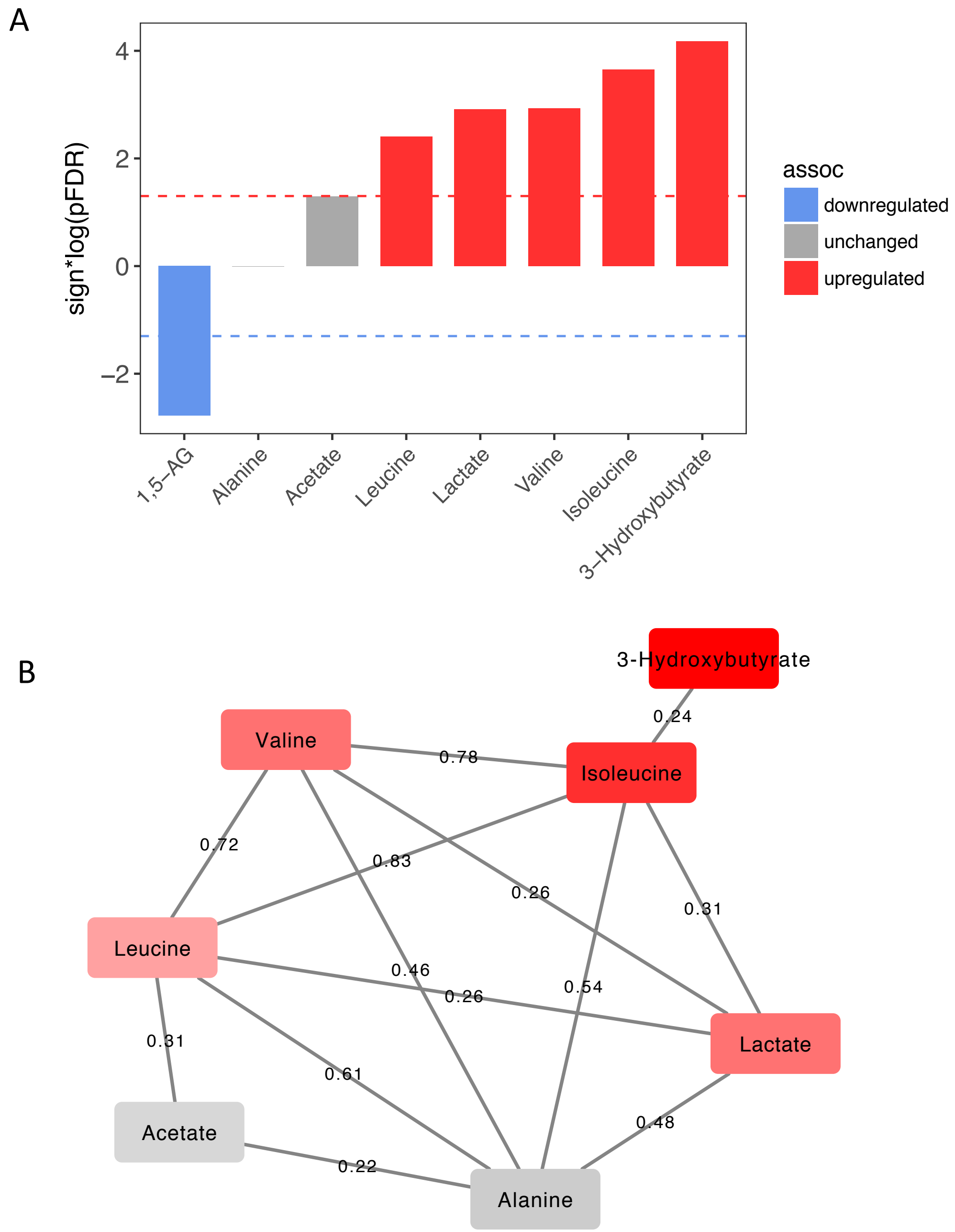

Figure S4. Visualization of the associations of type II diabetes (T2D) with 8 targeted plasma ^{1}H NMR metabolites in the FGENTCARD cohort (n = 506). The associations were computed using logistic regression models adjusted for age, gender, and body mass index (BMI). The *p*-values were adjusted for multiple-testing using BH-correction, with a significance threshold of 0.05. (A) Bar plot showing the $-\log_{10}$ (pFDR) x sign of beta coefficient of each metabolite. Statistically significant metabolites were colored in red if positively associated with T2D, and in blue if negatively associated with T2D. (B) Correlation-based metabolic network using a correlation threshold of r > |0.20|. Statistically significant nodes positively associated with T2D were colored in red, while not significant nodes were colored in gray.

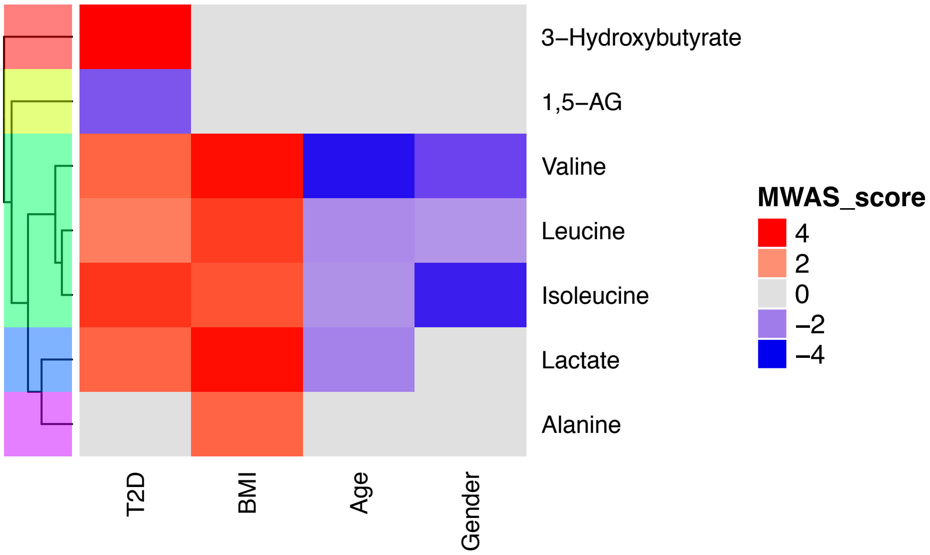

Figure S5. Visualization of the associations of multiple phenotypes with 8 targeted plasma ^{1}H NMR metabolites in the FGENTCARD cohort (n = 506). The associations for body mass index (BMI) and age were computed using Spearman partial correlations adjusted for gender, type II diabetes (T2D) and age (for the BMI models) or BMI (for the age models). The associations for T2D and gender were computed using logistic regression models adjusted for age, BMI and gender (for the T2D models) or T2D (for the gender models). The *p*-values were corrected for multiple-testing using BH-correction, with a significance threshold of 0.05. The heatmap shows the individual MWAS scores, defined as: $-\log_{10}$ (pFDR) x sign of estimate. Only metabolites significantly associated with at least one phenotype were displayed (*i.e.* acetate is not shown). The metabolites were ordered based on hierarchical cluster analysis of the auto-correlation metabolic matrix.

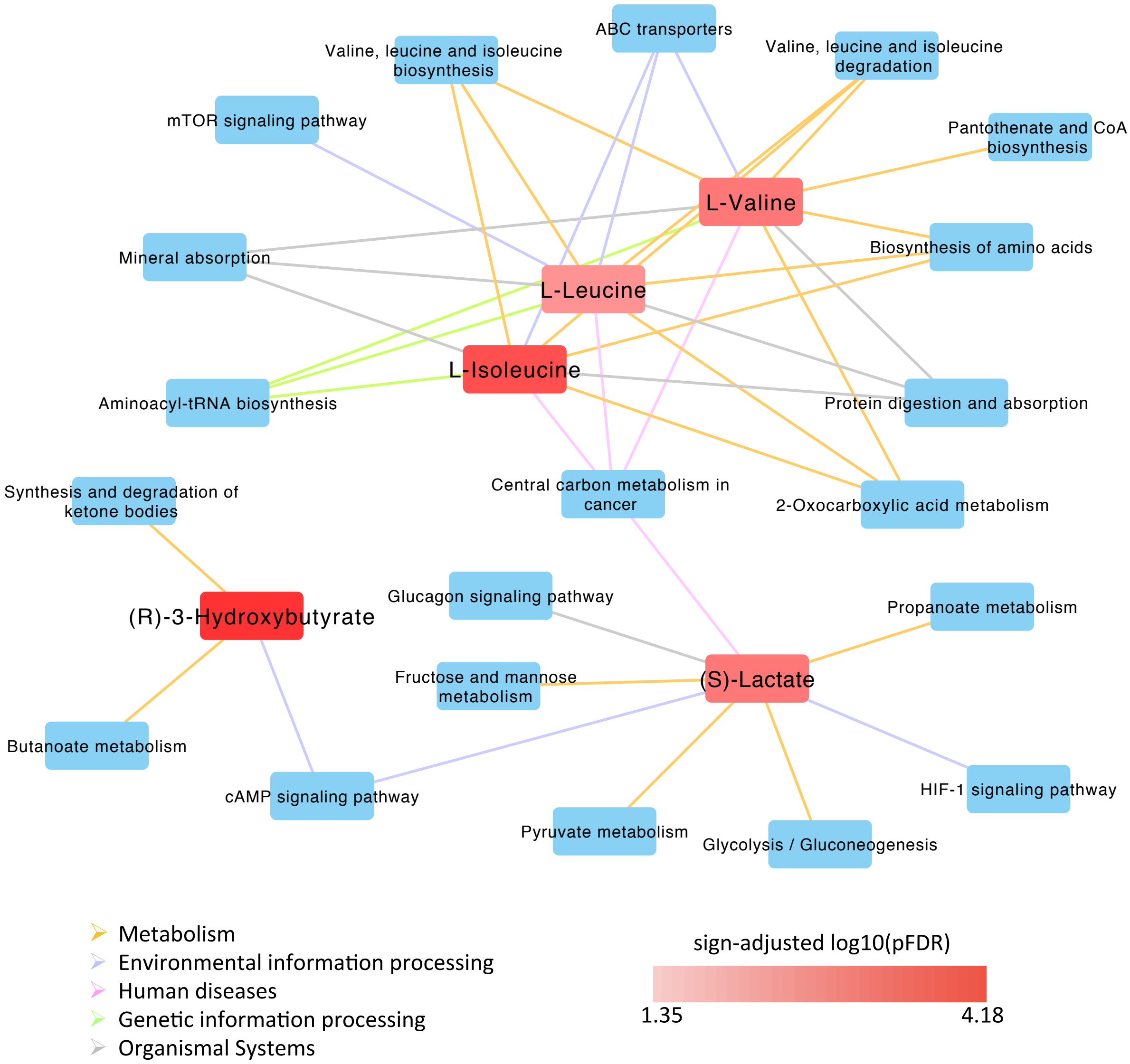

Figure S6. Mapping of metabolites significantly associated with type II diabetes onto the KEGG pathways. In total 5 of the significant targeted metabolites (Figure S4A) were mapped onto the KEGG pathways: lactate, leucine, isoleucine, valine and 3-hydroxybutyrate (1,5-AG is not reported in the KEGG pathways). The network shows the human KEGG pathways linked to each of the metabolites. The color of the metabolites reflects the degree of association with type II diabetes (*i.e.* $-\log_{10}$ (pFDR) x sign of beta coefficient), while the color of the edges indicates pathway class.

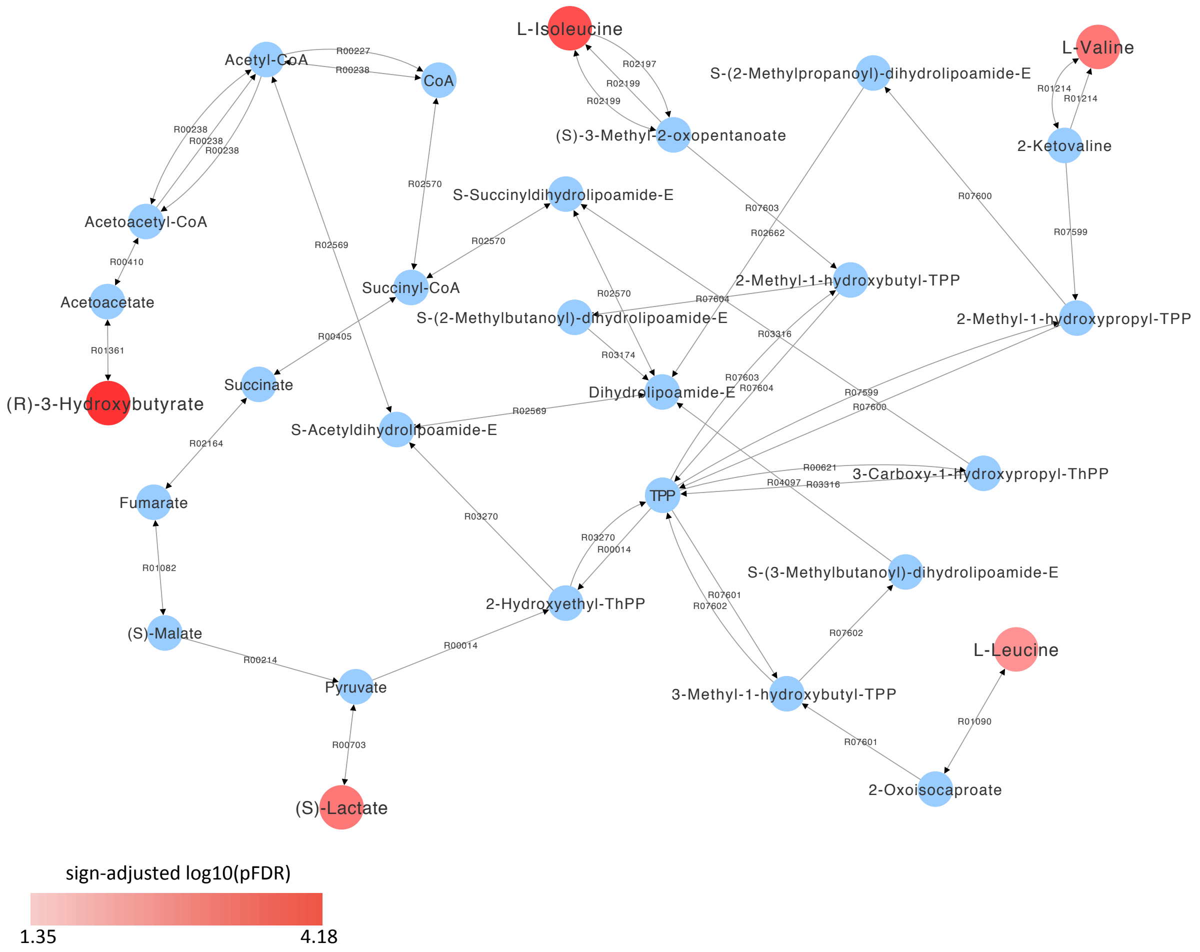

Figure S7. Representation of the biochemical relationships between the metabolites significantly associated with type II diabetes. In total 5 of the significant targeted metabolites (Figure S4A) were mapped onto the KEGG pathways: lactate, leucine, isoleucine, valine and 3-hydroxybutyrate (1,5-AG is not reported in the KEGG pathways). The network shows all shortest-paths between these 5 metabolites calculated from a global substrate-product network, comprising 51 relevant human metabolic pathways. The metabolites of interest were colored based on their degree of association with type II diabetes (*i.e.* $-\log_{10}$ (pFDR) x sign of beta coefficient). The reactions catalysing the metabolic conversions are shown in the edges. For simplification purposes, when there were several reactions linked to the same substrate-product pair, only one of them was shown in the network.

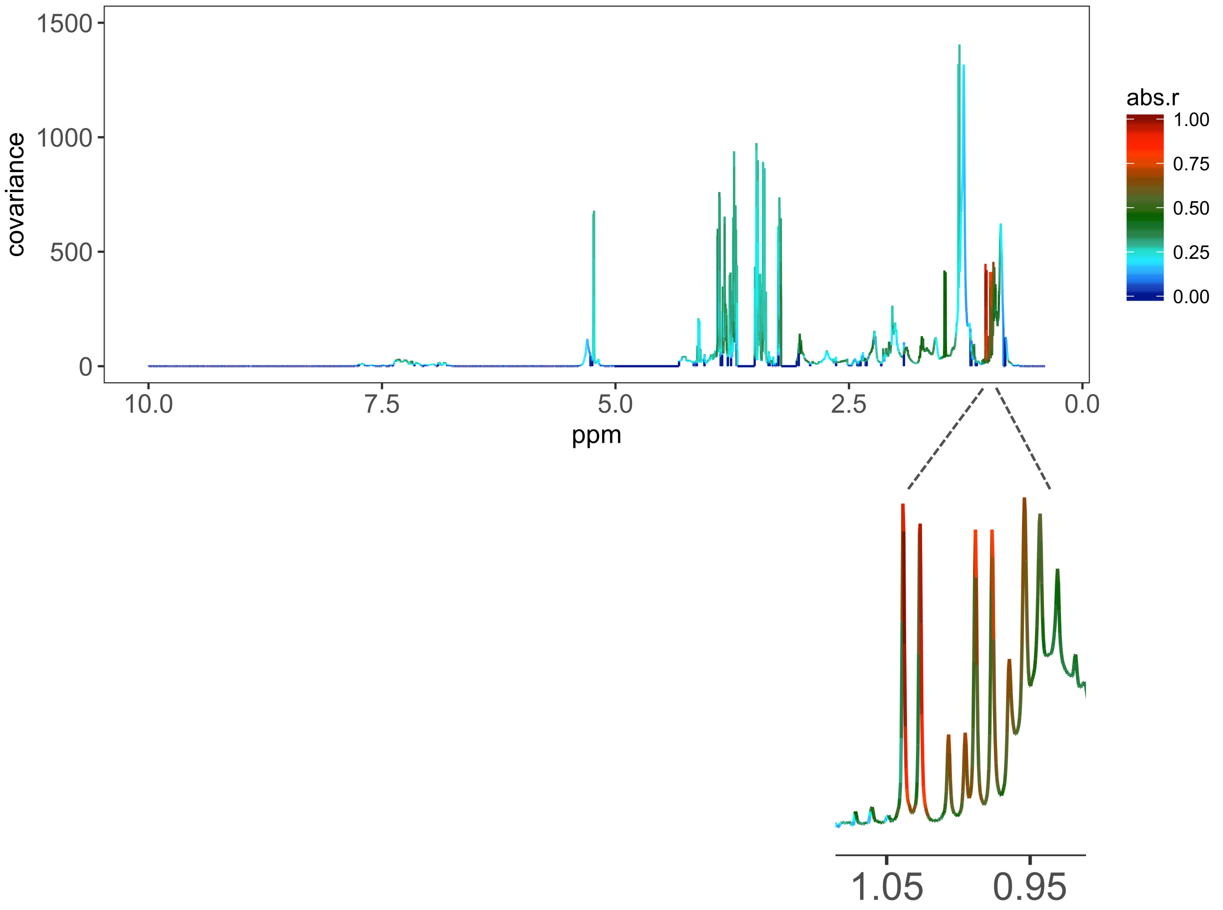

Figure S8. Visualization STOCSY results using 506 plasma ^{1}H NMR metabolic profiles from the FGENTCARD cohort, with δ 1.04 as driver signal. The results from STOCSY were visualized using a pseudo-NMR spectrum, showing the covariance (height) and correlation (color) of each NMR signal with the driver signal. In order to facilitate the identification of intramolecular correlations, only features significantly correlated (pFDR < 0.05) with the driver signal were displayed. The observed correlation pattern indicates that the driver signal corresponds to valine.
